# Exosomal Non-Coding RNAs in Pancreatic Cancer: From Mechanisms to Clinical Applications

**DOI:** 10.32604/or.2025.066150

**Published:** 2025-10-22

**Authors:** Chengru Yang, Zhiyu Wang, Shaowu Bi, Xinmiao Zhang, Zhaoqiang Xu, Yifei Ge, Tianjie Zhang, Nan Wang, Yi Xu, Xiangyu Zhong

**Affiliations:** 1 Department of Hepatopancreatobiliary Surgery, The Second Affiliated Hospital of Harbin Medical University, Heilongjiang, 150086, China; 2 State Key Laboratory of Targeting Oncology, National Center for International Research of Bio-targeting Theranostics, Guangxi Key Laboratory of Bio-targeting Theranostics, Collaborative Innovation Center for Targeting Tumor Diagnosis and Therapy, Guangxi Medical University, Nanning, 530021, China; 3 Key Laboratory of Functional and Clinical Translational Medicine, Fujian Province University, Xiamen Medical College, Xiamen, 361000, China; 4 Key Laboratory of Human Development and Disease Research (Guangxi Medical University), Education Department of Guangxi Zhuang Autonomous Region, Nanning, 530021, China; 5 Key Laboratory of Biological Molecular Medicine Research (Guangxi Medical University), Education Department of Guangxi Zhuang Autonomous Region, Nanning, 530021, China; 6 Fujian Provincial Key Laboratory of Tumor Biotherapy, Fuzhou, 350014, China; 7 Fujian Provincial Key Laboratory of Translational Cancer Medicine, Fuzhou, 350014, China; 8 School of Pharmacy, Anhui Engineering Technology Research Center of Biochemical Pharmaceutical, Bengbu Medical University, Bengbu, 223913, China; 9 Department of Pathology, Li Ka Shing Faculty of Medicine, The University of Hong Kong, Hong Kong, 999077, China

**Keywords:** Exosomes, non-coding RNAs, pancreatic cancer, biomarker, diagnosis, therapy

## Abstract

Pancreatic cancer (PC) is an extremely aggressive cancer of the digestive system with insidious onset and the lack of effective biomarkers, resulting in late-stage diagnosis and poor prognosis. Exosomal non-coding RNAs (ncRNAs) are key mediators of intercellular communication that drive PC initiation and advancement. By modulating gene expression, they impact tumor microenvironment (TME) remodeling, proliferation, migration, apoptosis, and immune evasion. Critically, exosomal ncRNAs serve as promising biomarkers for early diagnosis and prognostic assessment. This review summarizes the current research achievements regarding exosomal ncRNAs in PC, systematically elaborating on their roles in tumor occurrence, metastasis, chemoresistance and the TME. Furthermore, by integrating the potential of exosomal ncRNAs in the diagnosis, treatment and prognosis of PC and by highlighting the challenges and future directions, this review aims to offer novel insights for future research and clinical translation of exosomal ncRNAs in PC.

## 1 Introduction

Pancreatic cancer (PC) is one of the most fatal malignancies in the digestive system. Due to its highly aggressive nature, early metastasis, and late detection, its five-year survival rate is only 13%, making it the third leading cause of cancer-related death worldwide [1]. The primary risk factors associated with this disease include age, smoking, obesity, diabetes, and family history [2,3]. Most patients are diagnosed at an advanced stage, primarily owing to the lack of early clinical symptoms and specific biomarkers. Although various treatments, such as surgery, immunotherapy, and targeted therapy, have shown some progress, PC continues to exhibit poor responses to these interventions and has a high recurrence rate [4]. Therefore, an in-depth investigation of the molecular mechanisms underlying PC is essential for identifying new biomarkers for early diagnosis and for developing more effective treatment strategies.

Exosomes are a type of extracellular vesicles (EVs) secreted by cells and which are typically 40 to 160 nm in size enclosed by a lipid bilayer membrane. They carry various bioactive substances, comprising proteins, lipids and RNAs—especially non-coding RNAs. Exosomes are discharged from cells as a result of the fusion of multivesicular bodies (MVBs) with the cell membrane, a process that mediates intercellular communication and contributes to the regulation of physiological activities [5]. During the process of genome transcription, over 90% of RNA transcripts are not utilized for protein encoding. These non-translated RNAs, referred to as non-coding RNAs (ncRNAs), include microRNAs (miRNAs), long non-coding RNAs (lncRNAs), and circular RNAs (circRNAs) [6]. The delivery of ncRNAs via exosomes is essential for intercellular communication and significantly influences the tumor microenvironment (TME). Exosomal ncRNAs can influence tumor proliferation, metastasis, and chemoresistance [7]. Exosomal circATP8A1 derived from gastric cancer cells can regulate the miR-1-3p/STAT6 axis to induce macrophage M2 polarization, ultimately promoting proliferation and migration in gastric cancer [8]. Furthermore, metastatic breast cancer-derived exosomal miR-10b can induce invasive capabilities in non-invasive breast cells upon uptake [9]. Compared to traditional biomarkers, exosomal ncRNAs offer several advantages: (1) The lipid bilayer encapsulating exosomes protects ncRNAs from degradation, resulting in high stability; (2) The unique expression patterns of exosomal ncRNAs in tumors enable differentiation between tumor and healthy tissues, demonstrating high specificity; (3) The expression levels of certain exosomal ncRNAs correlate with tumor progression, including proliferation, metastasis and tumor stage, allowing for dynamic monitoring [10]. Although the mechanisms of exosomal ncRNAs are not fully understood, their association with cancer suggests their potential as early diagnostic biomarkers and new therapeutic targets for PC. The purpose of this review is to display the biological functions of exosomal ncRNAs and their regulation in PC. It thoroughly evaluates the clinical application potential of exosomal ncRNAs related to PC, and further identifies the challenges with clinical translation along with the future research directions, which provides reference for advancing the research on molecular mechanisms of PC and clinical translation.

## 2 The Biological Properties of Exosomes

In 1987, Johnstone’s team first identified the vesicles released during the maturation process of sheep reticulocytes as exosomes [11]. The generation of exosomes begins with the endocytosis of the plasma membrane, leading to the formation of early endosomes (EEs), which subsequently mature into MVBs. During the formation of MVBs, the membranes of endosomes indent to create intraluminal vesicles (ILVs), which will eventually become exosomes [12]. Finally, MVBs fuse with the cell membrane and release ILVs into the extracellular environment. The vesicles released into this environment are termed exosomes. It is important to highlight that the machinery known as the endosomal-sorting complex required for transport (ESCRT) is crucial in the sorting of proteins within ILVs [13,14].

As a crucial mediator of intercellular communication, exosomes primarily interact with receptor cells through three mechanisms: (1) uptake via endocytosis; (2) fusion with the receptor cell membrane; and (3) signaling through receptor-ligand binding at the cell membrane [15]. In malignant tumors, such as PC, exosomes significantly influence the TME. First, PC-derived exosomes function as messengers, transporting a diverse array of cargo, including ncRNAs, to various target cells. This process ultimately regulates the proliferation, invasion, and migration of PC cells by facilitating distinct signaling pathways [16]. Secondly, exosomes secreted by tumor cells can carry molecules, such as ncRNAs, and participate in the tumor’s immune evasion. For instance, exosomal circUSP7 secreted by non-small cell lung cancer (NSCLC) cells can inhibit CD8+ T cell function, thereby promoting immune evasion and resistance to anti-PD-1 treatment [17]. Furthermore, exosomes clearly contribute to angiogenesis within the TME. Tumor cell-derived exosomes can activate relevant signaling pathways that facilitate tumor angiogenesis, thereby supporting tumor growth and metastasis. Research has shown that gallbladder cancer cells-derived exosomes can transfer lncRNA TRPM2-AS to human umbilical vein endothelial cells (HUVECs), thereby activating the NOTCH1 signaling pathway and ultimately facilitating angiogenesis in gallbladder cancer [18]. Additionally, exosomes play a significant role in tumor drug resistance. Specifically, paclitaxel (PTX) promotes the release of exosomal circBACH1 in breast cancer cells, which contributes to drug resistance by reducing the levels of miR-217 and elevating the expression of GTPase-activating SH3 domain-binding protein 2 (G3BP2) [19]. To facilitate distant metastasis, exosomes enhance the shaping of the pre-metastatic niche (PMN). Specifically, ovarian cancer-secreted exosomal miR-141 activates the Yes-associated protein 1 (YAP1)/Growth-regulated oncogene-alpha (GROα)/Human C-X-C Motif Chemokine Receptors (CXCRs) signaling cascade, thereby enhancing the development of the PMN and ultimately leading to tumor metastasis [20].

The functional heterogeneity of exosomal ncRNAs is predicated on two primary aspects: (1) the selective sorting of ncRNAs into exosomes and (2) the targeted delivery of these exosomal ncRNAs to recipient cells. Firstly, as previously mentioned, the role of the ESCRT mechanism in protein sorting is crucial. The incorporation of ncRNAs into exosomes is not a random process; it is contingent upon a selective sorting mechanism. A classic study has demonstrated that the protein heterogeneous nuclear ribonucleoprotein A2B1 (hnRNPA2B1), an RNA-binding protein, specifically recognizes miRNAs containing short sequence motifs following sumoylation and facilitates their loading into exosomes [21]. Secondly, research has demonstrated that CD8+ dendritic cells specifically recognize ICAM-1 on exosomes derived from mature dendritic cells due to the high expression of LFA-1, facilitating the capture of functional MHC-peptide complexes and ultimately enabling the antigen transfer between dendritic cells [22]. This research illustrates that exosomes facilitate targeted delivery via specific receptor-ligand binding.

**Figure 1:**
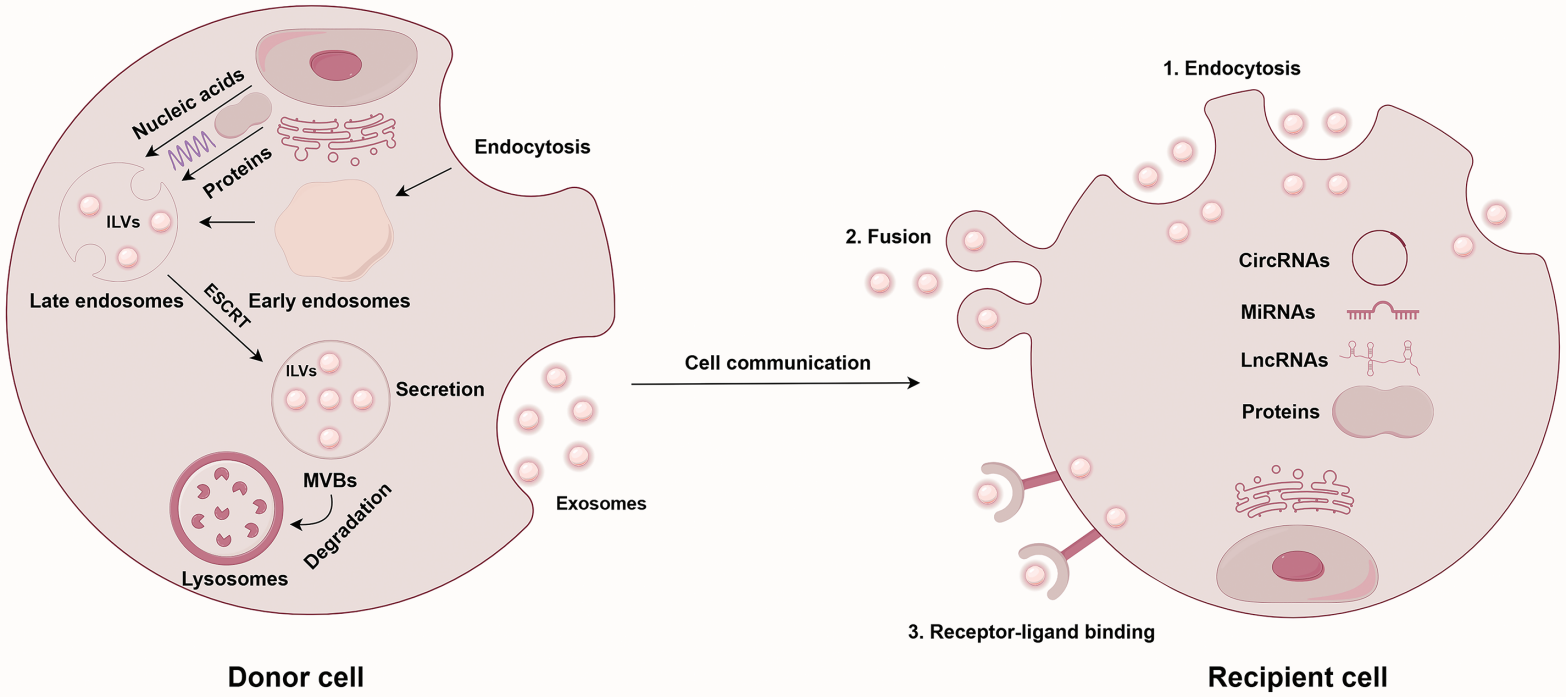
Exosome biogenesis and intercellular communication. Abbreviations: CircRNAs, circular RNAs; ESCRT, endosomal-sorting complex required for transport; ILVs, intraluminal vesicles; LncRNAs, long non-coding RNAs; MiRNAs, microRNAs; MVBs, multivesicular bodies. This figure is created by Figdraw (Copyright Code: AAOTR5aa5b)

In summary, exosomes act as crucial facilitators of intercellular communication and play a multifaceted role in the onset and progression of tumors. They transport signaling molecules, including ncRNAs, and significantly influence the TME by not only promoting tumor cell growth but also facilitating tumor immune evasion, angiogenesis, and drug resistance. These results highlight the crucial role of exosomes in oncology and offer new strategies for the precision treatment of tumors in the future. Fig. 1 shows the biosynthesis pathway of exosomes and their role in intercellular communication.

## 3 Types and Functions of ncRNAs

Initially regarded as “junk” transcripts, ongoing research has revealed that ncRNAs are essential in numerous biological processes, such as gene regulation and cell communication, particularly in the progression of malignant tumors [6]. Due to their diverse functions in regulating gene expression and intercellular communication in PC, ncRNAs are emerging as promising biomarkers and therapeutic targets; however, different ncRNAs exhibit slightly varied mechanisms. MiRNAs are short RNA fragments, approximately 22 nucleotides in length, that primarily inhibit or degrade mRNA translation by binding to the corresponding RNA-induced silencing complex (RISC), ultimately regulating the expression of specific genes [23]. In contrast, lncRNAs, which are longer than 200 nucleotides, can interact with proteins to modulate their functions. Notably, lncRNAs can act as “sponges” for miRNAs, utilizing the competing endogenous RNA (ceRNA) mechanism to diminish the targeting effect of miRNAs on mRNAs, thereby participating in the regulation of malignant tumor development [24]. Unlike miRNAs and lncRNAs, circRNAs possess a covalently closed ring structure, which confers greater stability due to their unique architecture. As a sponge for miRNA, circRNA not only engages with RNA-binding proteins to influence the biological behavior of malignancies but also takes part in various tumor-related signaling pathways, thereby modulating the cell cycle and proliferation [25]. A study has identified that the four miRNAs (hsa-miR-132-3p, hsa-miR-30c-5p, hsa-miR-24-3p, and hsa-miR-23a-3p) exhibit significant overexpression in early PC patients, indicating their potential utility in the early diagnosis of PC [26]. Beyond their specific expression, ncRNAs can also target and regulate PC cells, positioning them as promising new targets for treatment. Research has demonstrated that downregulating hsa_circ_0000069 can prevent the proliferation of PC cells by targeting the miR-144/SCL/TAL1 interrupting locus (STIL) axis. Furthermore, exosomes that downregulate hsa_circ_0000069 can prevent the malignant transformation of human pancreatic duct epithelial cells. Thus, targeting hsa_circ_0000069 represents a potential and feasible therapeutic strategy for PC [27]. Consequently, ncRNAs are essential in gene regulation and tumor development, significantly contributing to the onset and progression of tumors such as PC, and hold broad prospects for application in early diagnosis and targeted therapy.

## 4 The Role of Exosomal ncRNAs in Directly Regulating the Intrinsic Behaviors of PC Cells

### 4.1 Cell Proliferation, Migration & Invasion, Epithelial-Mesenchymal Transition (EMT)

Tumor proliferation, migration, and invasion are three key biological characteristics in the development of cancer. Growing evidence indicates that exosomal ncRNAs have a crucial regulatory function in the progression of PC. Under hypoxic conditions, hypoxia inducible factor 1α (HIF1α) can enhance the expression of hypoxic PC cells-derived exosomal circPDK1, which subsequently augments the proliferation and migratory capacity of PC cells [28]. The studies also identify that liver-metastatic PDAC cells-derived exosomal circ-PDE8A can enhance the invasive progression of PDAC cells through the miR-38/MACC1/MET axis. Notably, this exosomal RNA can enter the bloodstream, and its elevated expression in plasma is strongly linked to tumor aggressiveness and unfavorable outcomes in PDAC patients [29]. Recent research indicates that dying tumor cells-derived exosomal hsa_circ_0002130 can enhance the cell proliferation upon irradiation of PC cells through DNA damage repair mediated by the hsa_circ_0002130/hsa_miR_4482-3p/Nibrin (NBN) axis [30].

Epithelial-mesenchymal transition (EMT) is the process through which epithelial cells undergo a phenotypic change to become mesenchymal cells. While this process is essential for the formation of most normal tissues and organs, it also endows tumor cells with enhanced capabilities for progression and metastasis [31]. PC cells-derived exosomal long non-coding RNA regulator of reprogramming (lncROR) can induce adipocytes to dedifferentiate into preadipocyte/fibroblast-like cells. These dedifferentiated cells activate the HIF1α-ZEB1 signaling pathway by secreting interleukin-1b (IL-1b), thereby further promoting the development of PC cells, as well as enhancing their proliferation, migration, invasion capabilities, and accelerating EMT [32]. Additionally, melittin can stimulate the expression of lncRNA NONHSAT105177 in PDAC cell lines. Overexpression of NONHSAT105177 significantly downregulates the levels of various cholesterol biosynthesis genes and inhibits EMT-inducing transcription factors, which ultimately leads to the inhibition of growth, metastasis, and EMT in PDAC. In exosomes secreted by PDAC cells that overexpress NONHSAT105177, the expression level of this lncRNA is notably higher than that of the control group, suggesting its transport via exosomes [33]. Another PDAC cell-derived exosomal lncRNA Sox2ot, promotes the upregulation of Sox2 by competitively binding to the miR-200 family, thus enhancing the EMT capabilities and stem-like characteristics of PDAC cells [34]. Furthermore, pancreatic stellate cells (PSCs)-derived exosomes can activate the Ras/ERK and Ras/Akt signaling pathways through miR-21, further inducing EMT and enhancing the metastasis capacity of PC cells [35].

In conclusion, exosomal ncRNA significantly influences the progression of PC by targeting various signaling pathways. These studies not only elucidate the potential biological mechanisms underlying PC but also offer a valuable theoretical foundation for the development of targeted treatment strategies based on these pathways.

### 4.2 Chemoresistance

Drug resistance refers to the phenomenon whereby tumor cells lose sensitivity to therapeutic drugs during anti-tumor treatments, such as chemotherapy. It is a significant factor contributing to the unfavorable prognosis of patients with PC. Exosomes, as crucial mediators of intercellular communication, not only directly influence the drug sensitivity of tumor cells by transporting ncRNAs, including miRNA, lncRNA, and circRNA, but also facilitate the dissemination of chemoresistance to neighboring cells, thereby creating a chemoresistant microenvironment. For instance, studies have shown that elevated levels of miR-155 can induce chemoresistance to gemcitabine (GEM) in PDAC cells and enhance the secretion of exosomes from GEM-resistant PDAC cells. These exosomes transfer miR-155 to neighboring cancer cells, leading to the development of GEM chemoresistance in those cells and further increasing exosome secretion [36]. Additionally, macrophage-derived exosomes can convey miR-365 to PC cells, where miR-365 promotes GEM chemoresistance by upregulating triphosphate nucleotide pool levels and enzyme cytidine deaminase activity [37]. Furthermore, miR-210 is significantly upregulated in exosomes secreted by GEM-chemoresistant PC stem cells and is transferred to GEM-sensitive PC cells via exosomes, significantly augmenting their chemoresistance. Mechanistically, exosomal miR-210 enhances the phosphorylation levels of mammalian target of rapamycin (mTOR) and S6K1, a downstream target of mTOR, thereby activating the mTOR pathway, which counteracts GEM-induced cell cycle arrest and apoptosis, ultimately facilitating the horizontal transfer of drug-resistant traits in PC [38]. Notably, circZNF91-rich exosomes released from hypoxic PC cells have the capability to be transferred to PC cells in a normoxic environment. CircZNF91 further promotes the upregulation of Sirtuin1 (SIRT1) expression, stabilization of HIF-1α protein, and enhancement of glycolytic activity by acting as a molecular sponge for miR-23b-3p, which leads to GEM chemoresistance [39]. Additional studies have demonstrated that PC cells-derived exosomes, prompted by hypoxia, hinder the activation of the Hippo/Yes-associated protein (YAP) signaling pathway by transporting lncROR, thereby increasing the stemness of PC cells and chemoresistance to GEM. Specifically, hypoxia-induced tumor-derived exosomal lncROR can prevent GEM-induced apoptosis and cell cycle arrest, significantly improving the chemoresistance of PC to GEM [40].

In summary, exosomes derived from various cell types play a crucial role in the development and dissemination of chemoresistance in PC by transporting a diverse array of ncRNAs. A thorough investigation into the mechanisms by which exosomal ncRNAs contribute to chemoresistance in PC not only enhances our understanding of the intricate molecular network associated with this disease but also offers novel research avenues for addressing the challenges of chemotherapy resistance and treatment strategies in PC. Table 1 shows the regulatory role of exosomal ncRNAs on the intrinsic behavior of PC cells.

**Table 1:** The role of exosomal ncRNAs in pancreatic cancer

Donor cells	ncRNAs	Pathway	Functions	References
Hypoxia PC cells	circPDK1	Modulate miR-628-3p/BPTF axis and degrade BIN1	Promote proliferation and migration	[28]
Liver-metastatic PDAC cells	circPDE8A	Modulate miR-338/MACC1/MET pathway	Promote proliferation and invasion	[29]
Human PC cells upon irradiation	hsa_circ_0002130	Modulate hsa_circ_0002130/hsa_miR_4482-3p/NBN axis to promote DNA damage repair	Promote cell proliferation upon irradiation	[30]
PC cells	linc-ROR	Induce adipocytes to dedifferentiate into preadipocyte/fibroblast-like cells then activate the HIF1α-ZEB1 signaling pathway	Enhance proliferation, migration, invasion and accelerate EMT	[32]
PDAC cells	lncRNA NONHSAT105177	Downregulate the cholesterol biosynthesis pathway	Inhibit the growth, migration and EMT	[33]
PDAC cells	lnc-Sox2ot	Competitively bind miR-200 and regulate Sox2 expression	Promotes EMT and stemness	[34]
Pancreatic stellate cells	miR-21	Activate the Ras/ERK and Ras/Akt signaling pathways	Promote EMT and metastasis	[35]
GEM-resistant PDAC cells	miR-155	Induce exosome secretion and chemoresistance	Promote GEM resistance	[36]
Macrophages	miR-365	Upregulate the triphospho-nucleotide pool and induce enzyme cytidine deaminase activity	Promote GEM resistance	[37]
PC stem cells	miR-210	Deliver miR-210 and mediate horizontal transfer of GEM-resistant traits	Promote GEM resistance	[38]
Hypoxic PC cells	circZNF91	Sponge miR-23b-3p to elevate SIRT1 expression and stabilize HIF-1α	Promote GEM resistance of normoxic PC cells	[39]
Hypoxic PC cells	lncROR	Inactivate Hippo/YAP pathway	Promote GEM resistance and stemness	[40]
NK cells	microRNA-let-7b-5p	Negatively regulates CDK6	Inhibit proliferation	[41]

Note: EMT, epithelial-mesenchymal transition; GEM, gemcitabine; HIF1α, hypoxia inducible factor 1α; LncROR, long non-coding RNA regulator of reprogramming; NBN, Nibrin; NK cells, natural killer cells; PC, pancreatic cancer; PDAC, pancreatic ductal adenocarcinoma; YAP, Yes-associated protein.

## 5 The Functions of Exosomal ncRNAs in Modifying the TME and Facilitating Metastasis in PC

In addition to the effects on the intrinsic behaviors of PC cells discussed in the Section 4, exosomal ncRNAs play crucial roles in remodeling the TME. These preparations for metastasis and taking part in various signaling pathways which ultimately support tumor growth. This section discusses exosomal ncRNAs-mediated functions that have been reported in the research.

### 5.1 The Influence of Exosomal ncRNAs on Immune Cells

Exosomes serve as a crucial information transmission medium between tumor cells and immune cells, regulating the functions and responses of immune cells through the ncRNAs they carry. An increasing amount of research has shown that exosomal ncRNAs influence immune cell activity, immune tolerance, and macrophage polarization, thereby modulating the process of tumor immune evasion [42,43]. Investigating the regulatory effects of exosomal ncRNAs on immune cells offers novel insights into the mechanisms underlying PC immune escape and the development of immunotherapy strategies.

Exosomes secreted by PC cells carry miR-203, which is taken up by dendritic cells and leads to a significant downregulation in toll-like receptor (TLR) 4 protein expression. This downregulation hinders the release of tumor necrosis factor-α (TNF-α) and IL-12, thereby weakening the antigen presentation and immune function of dendritic cells, ultimately promoting immune evasion in the PC microenvironment [44]. Natural killer (NK) cells are also influenced by the PC microenvironment. Studies have demonstrated that NK cell exosomes transfer microRNA-let-7b-5p to PC cells, targeting and regulating the cell cycle gene CDK6, which inhibits tumor cell proliferation. However, following co-culture with PC cells, the miRNA content and cytotoxicity in NK cell-derived exosomes decrease, indicating that PC cells may regulate the levels of miRNA in NK cell exosomes and diminish their anti-tumor function, contributing to immune evasion [41]. Additionally, EVs secreted by cancer-associated fibroblasts (CAFs) contain lncRNA RP11-161H23.5, which can be transported to PC cells where it forms a complex with CNOT4, leading to the downregulation of MHC I, particularly human leukocyte antigen (HLA)-A expression levels. This process thereby promotes the immune evasion of PC cells [45].

In summary, exosomes derived from PC and other cells carry ncRNAs, interfere with immune cell function through various pathways, promote immune evasion, and consequently contribute to the progression of PC. These findings not only enhance our understanding of the immune microenvironment associated with PC but also offer new treatment strategies and possibilities for overcoming the immune escape mechanisms of this malignancy.

### 5.2 The Role of Exosomal ncRNAs in Tumor Angiogenesis

Angiogenesis serves as a critical link within the TME, supplying essential oxygen and nutrients for tumor growth and dissemination. Exosomal ncRNAs have been identified as a promoter of the malignant progression of tumors through their regulation of angiogenesis-related signaling pathways [46,47]. A thorough comprehension of the regulatory role of exosomal ncRNAs in PC angiogenesis could unveil new perspectives on the pathogenesis of this disease and facilitate the exploration of novel treatment approaches.

In hypoxic environments, exosomes from PC cells exhibit a notable enrichment of miR-30b-5p, which is then conveyed to endothelial cells. This miRNA promotes angiogenesis by suppressing the production of the gap junction protein GJA1 [48]. Additionally, lncRNA UCA1 exhibits high levels in exosomes from hypoxic PC cells and is delivered to HUVECs via these exosomes. UCA1 enhances angiogenesis by improving the migration and lumen formation abilities of HUVECs. Its mechanism involves functioning as a ceRNA for miR-96-5p, promoting the expression of AMOTL2, and consequently increasing the levels of p-ERK1/2 [49].

In conclusion, exosomes are crucial in the angiogenesis of PC as they transport ncRNAs to endothelial cells, which in turn modulate the expression of relevant proteins. These studies have improved the comprehension of PC angiogenesis and have provided novel perspectives for treatment approaches focused on angiogenesis in PC.

### 5.3 The Effect of PC Cell-Derived Exosomes on Distant Organs and Tissues

PMN refers to the establishment of a specific site conducive to metastasis, characterized by a series of molecular and organ changes, effectively creating the ‘soil’ necessary for the colonization of metastatic tumor cells [50]. This process facilitates the settlement of tumors in distant organs and promotes tumor metastasis. Increasing evidence indicates that a diverse array of bioactive molecules, including ncRNAs, transported by exosomes, can facilitate the formation of PMN, thereby promoting tumor metastasis [51]. Investigating the precise role of exosomal ncRNAs and other molecules in the impact of PC on distant organs and tissues is anticipated to enrich our comprehension of the processes involved in the progression and metastasis in PC, potentially leading to new avenues for innovative treatment strategies.

Studies have demonstrated that pancreatic adenocarcinoma cells-derived exosomes are abundant in miR-494 and miR-542-3p. These two miRNAs can target the expression of cadherin-17 (CDH17) and upregulate levels of matrix metalloproteinase (MMP) in target cells. Through this mechanism, PC-derived exosomes can specifically target untransformed cells in pre-metastatic organs, regulating these cells via miRNA to adapt to the requirements of tumor cell metastasis [52]. Additionally, the tRNA fragment tRF-GluCTC-0005 found in PDAC-derived exosomes can enhance the stability of WD repeat-containing protein 1 (WDR1) mRNA and activate hepatic stellate cells, thereby mediating the infiltration of myeloid-derived suppressor cells, promoting the formation of PMN, and facilitating liver metastasis of PC [53]. The study also reveals that EVs derived from CAFs are rich in PNI-associated transcript (PIAT), which is closely associated with perineural invasion (PNI) in PDAC patients. Mechanistically, PIAT inhibits the interaction between Y box binding protein 1 (YBX1) and neural precursor cell expressed, developmentally down-regulated 4-like (Nedd4l), stabilizing YBX1 and driving the perineural invasion of PC cells in a 5-methylcytosine (m5C)-dependent manner [54].

All in all, exosomal ncRNAs in PC regulate distant organs and tissues to adapt to the needs of tumor cell metastasis, ultimately promoting the formation of PMNs and metastasis. An in-depth exploration of the role of exosomal ncRNAs in metastasis will enhance our understanding of the mechanisms underlying PC metastasis and provide crucial theoretical guidance for the development of therapeutic strategies aimed at preventing this process.

### 5.4 The Crosslinks between Exosomal ncRNAs and Other Mechanisms

In addition to influencing immune evasion, angiogenesis, and distant tissues and organs, exosomal ncRNAs also interact with CAFs and are involved in the regulation of ferroptosis, metabolic reprogramming, and other mechanisms that contribute to malignant progression. Exosomal ncRNAs play an indispensable role in various prominent pathways through complex molecular networks, ultimately promoting tumor progression [55–57]. Elucidating the regulatory role of exosomal ncRNAs in these pathways is of great significance for a deeper understanding of the pathological mechanisms underlying PC, as well as for the exploration of new therapeutic targets.

As a critical component of TME, CAFs not only support tumor proliferation and metastasis through various mechanisms but also play essential roles in processes such as tumor drug resistance, immune evasion, and reactivation from tumor dormancy, thereby influencing tumor growth in multiple dimensions [58]. Furthermore, exosomes secreted by PC cells are rich in miR-1246 and miR-1290. Subsequent research has demonstrated that elevated levels of miR-1290 can enhance the expression of profibrogenic genes, induce the activation of PSCs, and augment profibrogenic activities, thereby contributing to the formation of the TME in PC [59]. Ferroptosis, a distinct iron-dependent non-apoptotic cell death that relies on iron and is marked by iron accumulation, reactive oxygen species (ROS) production, and lipid peroxidation, significantly influences the progression and treatment of various tumors, including PC [60,61]. Among the key players in this process is acyl-CoA synthetase long-chain family member 4 (ACSL4), which is essential for lipid peroxidation [62]. Research indicates that miR-3173-5p, abundant in exosomes derived from CAFs in PC, can inhibit ferroptosis in PC cells, ultimately promoting chemoresistance. Mechanistically, following GEM treatment, exosomal miR-3173-5p from CAFs enhances resistance to ferroptosis by inhibiting ACSL4, thereby increasing GEM chemoresistance in PDAC cells [63]. Metabolic reprogramming, a hallmark of malignant tumors, dynamically regulates metabolic pathways to fulfill the needs of tumor cells, enabling them to adapt to their environment and support specific functions. This process is essential for the development and therapy of PC [64,65]. In PDAC cells, exosomal lncRNA Nicotinamide nucleotide transhydrogenase-antisense RNA1 (NNT-AS1) derived from CAFs acts as a molecular sponge for miR-889-3p. This miRNA can target HIF-1α, ultimately promoting glucose metabolism reprogramming and the progression of PDAC [66]. Type 2 diabetes mellitus (T2DM) poses a considerable risk for PC, with insulin resistance and the resulting hyperinsulinemia serving not only as key features of T2DM but also as critical contributors to the malignant progression and unfavorable prognosis of PC [67,68]. Studies have demonstrated that PC cells-derived exosomes promote insulin resistance in skeletal muscle cells via the PI3K/Protein kinase B (Akt)/FoxO1 signaling pathway. Further microarray analysis has indicated that exosomal miRNA may be a key promoter of the disruption of skeletal muscle metabolic balance in PC [69].

**Figure 2:**
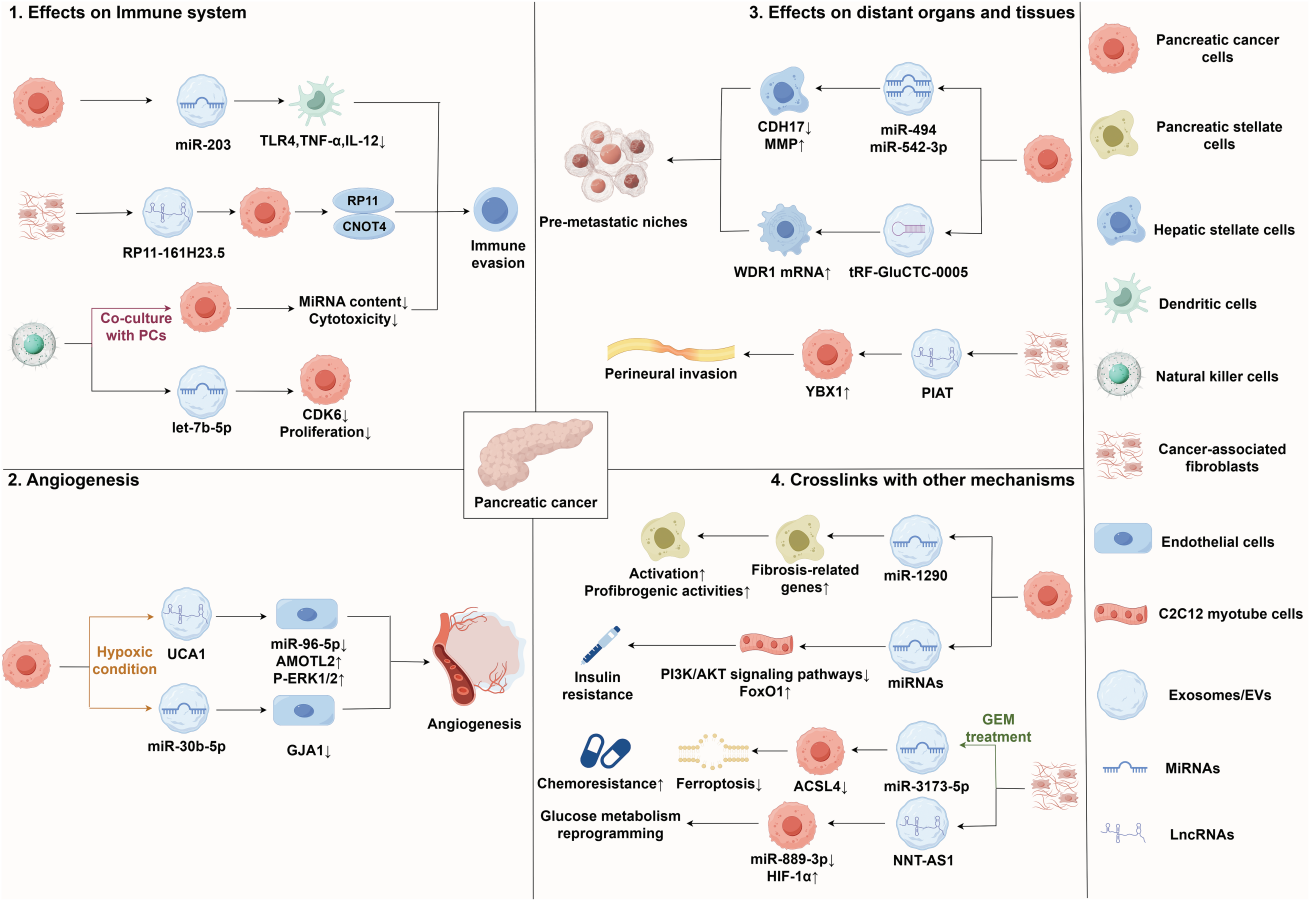
Interrelationship between exosomal ncRNAs and TME of PC. Abbreviations: ACSL4, acyl-CoA synthetase long-chain family member 4; CDH17, cadherin-17; HIF1α, hypoxia inducible factor 1α; MMP, matrix metalloproteinase; PCs, pancreatic cancer cells; PIAT, perineural invasion-associated transcript; TNF-α, tumor necrosis factor-α; TLR, toll-like receptor; WDR1, WD repeat-containing protein 1; YBX1, Y box binding protein 1. This figure is created by Figdraw (Copyright Code: IWRSA29823)

In conclusion, exosomal ncRNAs are vital in the TME and the development of PC by modulating various signaling pathways, which encompass the functions of CAFs, ferroptosis, and metabolic reprogramming. These studies, which address contemporary mechanisms, not only clarify the intricate nature and importance of exosomal ncRNA in the progression of PC but also present novel research directions and therapeutic targets for treatment approaches against this aggressive disease. Fig. 2 illustrates the interrelationship between exosomal non-coding RNAs and the TME of PC.

## 6 The Clinical Prospects of Exosomal ncRNAs in PC

### 6.1 Biomarkers for Early Diagnosis of PC

Exosomal ncRNAs are essential for the onset and evolution of malignancies, exhibiting abnormal expression in the bodily fluids of affected individuals. Furthermore, the protective properties of the lipid bilayer membrane, combined with the widespread presence of exosomal ncRNAs in body fluids, contribute to its high stability and ease of detection in liquid biopsies. These properties render exosomal ncRNAs a non-invasive biomarker, offering new insights and directions for the early clinical diagnosis of various cancer types [70,71]. A thorough comprehension of the expression characteristics and diagnostic accuracy of exosomal ncRNAs in PC is anticipated to offer new insights and directions for the early clinical diagnosis of this disease.

Research conducted on 32 individuals diagnosed with PC and 29 individuals with intraductal papillary mucinous neoplasm (IPMN) demonstrates that, in comparison to 22 controls without malignant or neoplastic lesions, the expression levels of exosomal miR-191, miR-21, and miR-451a are notably upregulated. The diagnostic abilities of these exosomal miRNAs in identifying IPMN and early PC surpass those of conventional markers like carcinoembryonic antigen (CEA) and cancer antigen 19-9 (CA19-9). Additionally, exosomal miR-191, miR-21, and miR-451a exhibit greater sensitivity in diagnosing IPMN and PC than extracellular circulating miRNAs found in serum or plasma [72]. Through bioinformatics analysis and cellular experiments, the researchers identify seven exosomal miRNAs (miR-31-5p, miR-31-3p, miR-210-3p, miR-339-5p, miR-425-5p, miR-425-3p, and miR-429) that are expressed differently when comparing PC cell lines to the control group, which consists of a cultured model of immortalized pancreatic duct epithelial cells. These miRNAs demonstrate potential diagnostic value. Furthermore, significant expression differences are observed between chronic pancreatitis and PDAC, offering advantages for monitoring high-risk patients [73]. A study utilizing a tethered cationic lipoplex nanoparticle (TLCN) biochip to detect exosomal miRNAs in peripheral blood (PB) reveals that, compared to healthy controls, PC patients exhibit markedly increased levels of exosomal miR-21 and miR-10b [74]. Further analysis using combined receiver operating characteristic (ROC) curves indicates that exosomal miR-10b enhances the diagnostic performance of exosomal miR-21. Notably, exosomal miR-21 demonstrates significant differences in expression when comparing patients with early-stage PC to healthy controls as well as to those with advanced-stage PC. This research offers a novel non-invasive method for the early diagnosis of PC [74]. Additionally, studies integrating Exosome Next-Generation Sequencing (EXO-NGS) and quantitative reverse transcription PCR (qRT-PCR) technology have shown that the levels of lncRNA MALAT1 and CRNDE in serum exosomes from individuals with PDAC and IPMN were higher than those in healthy samples, underscoring the potential value of exosomal ncRNAs in differentiating between healthy and malignant pancreatic conditions [75]. A different investigation reveals that the combined application of miR-200b and miR-200c in total serum exosomes, as well as in epithelial cell adhesion molecule (EpCAM)-positive serum exosomes alongside CA19-9, can significantly enhance the diagnostic accuracy for PDAC. Furthermore, miR-200b derived from EpCAM-positive serum exosomes is linked to shorter patient survival and may act as an independent prognostic indicator for PDAC [76]. A study has utilized genome-wide profiling to establish a set of cell-free and exosomal miRNA biomarkers for the early detection of PC. Subsequent experiments have validated that this transcriptomic signature based on exosomes, comprising 5 cell-free and 8 exosomal miRNAs in plasma, can effectively distinguish early-stage PDAC patients and demonstrates strong diagnostic performance. For both all PDAC patients and those with early-stage PDAC, the area under the curve (AUC) value of CA19-9 combined with this transcriptomic signature exceeds that of CA19-9 alone. Furthermore, this transcriptomic signature can effectively identify 20 patients with negative CA19-9 levels among 22 PDAC patients. This liquid biopsy-based diagnostic approach opens new avenues for the early detection of PDAC and is anticipated to complement traditional biomarkers [77]. Although previous studies have demonstrated that exosomal ncRNAs possess the potential to serve as novel biomarkers for PC, several limitations remain. The lncRNAs encapsulated within EVs derived from CAFs may facilitate immune evasion in PC; however, there are significant gaps in understanding the molecular mechanisms involved, unverified long-term biological safety and challenges related to clinical translation [45]. Furthermore, while exosomal tRFs have been associated with the promotion of liver metastasis, issues persist, such as the reliance on a single diagnostic marker, the inability of mouse models to fully replicate the complex TME of human PC and a lack of comprehensive mechanistic insights [53].

To sum up, exosomal ncRNAs demonstrate significant potential for the early detection of PC and related diseases. Furthermore, its diagnostic performance has shown advantages over traditional tumor markers in research, effectively distinguishing between PC patients, healthy individuals, and those with alternative pancreatic diseases. By integrating multiple detection technologies, the combined applications of exosomal ncRNAs enhance the accuracy of PC diagnosis. These research findings provide crucial insights for the early screening of PC and establish a strong foundation for developing exosomal ncRNA as novel biomarkers.

### 6.2 Therapeutic Target for PC

Numerous studies have demonstrated that exosomal ncRNAs not only influence the proliferation, invasion, and chemoresistance of PC cells but also impact the TME, positioning it as a promising point for PC therapy. Exosomal ncRNAs are fundamental in intercellular communication and gene regulation. By influencing relevant signaling pathways, it not only offers new insights for the treatment of tumors but also establishes a foundation for the advancement of precision medicine [78,79]. Therefore, further exploration of the clinical utility of exosomal ncRNAs in the treatment of PC will provide significant theoretical support and clinical guidance for enhancing the therapeutic effects and outcomes of patients with PC.

The research reveals a notable reduction in the expression level of miR-485-3p in PC tissues, while this miRNA is found to be enriched in exosomes sourced from pancreatic ductal epithelial cells. As a direct target of miR-485-3p, p21-activated kinase-1 (PAK1) exerts an inhibitory effect on PC cells. Notably, normal pancreatic ductal epithelial cells-derived exosomes can transfer miR-485-3p to PC cells, thereby inhibiting metastasis through direct targeting of PAK1. Consequently, utilizing exosomes for the delivery of miR-485-3p may represent a new therapeutic approach for PC [80]. Furthermore, miR-145-5p demonstrates significant inhibitory effects on the proliferation and invasion of PDAC cells. Exosomes have been successfully engineered from human umbilical cord-derived mesenchymal stromal cells (hucMSCs) to serve as carriers for transporting miR-145-5p into PDAC cells, effectively inhibiting cell proliferation and invasion while fostering apoptosis and inducing cell cycle arrest. This innovative approach offers a promising new direction for treating individuals diagnosed with PDAC [81]. Additional research has shown that exosomal miR-1231 derived from bone marrow mesenchymal stem cells (BM-MSCs) can impede the proliferation of PC cells, as well as negatively regulate the migration, invasion, and adhesion of the matrix of PC cells. Furthermore, reduced concentrations of exosomal miR-1231 in PB are closely associated with the TNM stage of PC. This finding suggests that high levels of exosomal miR-1231 derived from BM-MSCs may serve as a novel and effective therapeutic agent for PC BM-MSCs can act as an innovative and effective treatment option for PC, while PB exosomal miR-1231 may act as an important diagnostic biomarker for the disease [82]. CAF-derived EV-packaged circBIRC6 enhances the chromatin localization of XRCC4 through SUMOylation, which contributes to the emergence of oxaliplatin resistance in PC. Research conducted on patient-derived xenograft (PDX) models from patients resistant to oxaliplatin reveals that reducing circBIRC6 levels using antisense oligonucleotide (ASO) or in combination with olaparib significantly improves the response to chemotherapy. This finding offers a novel approach to overcoming platinum resistance in PC [83].

Collectively, these findings indicate that exosomal ncRNAs hold significant promise for the precise therapy of PC. Future research aimed at targeting relevant signaling pathways to develop novel therapeutic agents, along with the creation of exosome-based ncRNAs delivery tools, is anticipated to yield new breakthroughs in the precise management of PC, ultimately enhancing clinical outcomes for patients.

### 6.3 Biomarker for Evaluating Therapeutic Efficacy and Prognosis of PC

In addition to serving as early diagnostic markers and therapeutic targets, exosomal ncRNAs significantly contribute to monitoring the therapeutic effects of various malignancies. The ncRNAs carried by exosomes are not only highly accurate and specific, but also accurately reflect the biological characteristics of cancer cells [84,85]. Recent research has shown that the analysis of exosomal ncRNAs can effectively evaluate the therapeutic response in PC and assess prognosis, thereby providing valuable guidance for the development of personalized treatment plans.

A study involving 55 patients with PDAC following radical pancreatectomy found that the expression levels of exosomal miR-4525, miR-451a, and miR-21 in portal venous blood (PVB) and PB are linked to a heightened risk of postoperative recurrence, which is closely related to poor prognosis. Notably, the sensitivity, specificity, and accuracy of these exosomal miRNAs in PVB are observed to surpass those in PB, suggesting that exosomal miR-4525, miR-451a, and miR-21 in PVB can function as promising biomarkers for determining poor prognosis in post-surgical PDAC patients [86]. Additionally, some studies have reported that plasma exosomal miR-451a shows a significant upregulation in PDAC, as determined through miRNA microarray analyses. Further investigations indicate that exosomal miR-451a not only demonstrates a significant association with recurrence and unfavorable prognosis in PDAC patients but also constitutes a crucial independent prognostic indicator for overall survival (OS) and disease-free survival (DFS) rates. Importantly, a significant statistical relationship is observed between the expression level of exosomal miR-451a and both tumor size and stage. These findings indicate that plasma exosomal miR-451a can serve as a biomarker for both recurrence and prognosis in PDAC, highlighting its significant potential to become a clinically effective detection tool in the future [87]. Another study examined 210 plasma and serum samples from four groups of PDAC patients and developed a blood-derived exo-miRNA panel (EMP) that comprises six specific exosome miRNAs. Following further experimental validation, the EMP has been shown to serve as a separate predictor of recurrence-free survival (RFS) in patients with PDAC and also possesses predictive value for those undergoing neoadjuvant therapy (NAT). Moreover, the combined detection of EMP and CA19-9 significantly enhances the accuracy of prognosis predictions in PDAC patients. This study demonstrates that EMP can reliably predict the recurrence risk of PDAC patients after surgery and evaluate the potential risk of poor prognosis in those receiving NAT, thereby aiding clinicians in making more informed decisions [88]. Additionally, the expression of exosomal circ-IRAS is elevated in PC cells. Exosomes originating from PC can transfer circ-IRAS to HUVECs, promoting tumor invasion and metastasis. Further analysis indicates a positive relationship between the expression level of circ-IRAS and tumor metastasis, while also showing a negative relationship with patient survival duration. These findings suggest that exosomal circ-IRAS can be a significant biomarker for monitoring the prognosis of PDAC patients [89].

Taken together, exosomal ncRNAs demonstrate significant potential for monitoring therapeutic effects and evaluating the prognosis of PC. A comprehensive investigation into the characteristics of exosomal ncRNAs and their role in PC development is expected to yield more precise prognostic indicators, support better personalized treatment strategies for this disease, and ultimately improve patient quality of life.

### 6.4 Challenges and Future Prospects

The exosomal ncRNAs show a huge potential for application in diagnosis and therapy of PC; however, certain obstacles hinder their clinical application. There are challenges in the standardization of exosome isolation, efficiency of delivery of ncRNAs and biocompatibility of exosome-based therapy. These problems need to be tackled for effective promotion of this technology in clinical applications.

Ultra-speed centrifugation, as a gold standard for exosome isolation, has benefits such as low cost, suitability for large-scale preparation and high purity product. However, it is associated with high equipment requirements and extended processing times. Ultrafiltration takes less time and uses less equipment, yet it suffers from challenges such as membrane blockage, which can lead to raw material loss and potential deterioration caused by shear stress. Size-exclusion chromatography preserves the natural activity of exosomes and exhibits good repeatability; however, its high cost and the necessity for additional exosome enrichment methods impede its commercial use. Polymer precipitation is advantageous due to its convenience and efficiency, but it also encounters issues related to contaminant presence and prolonged processing times. Immunoaffinity capture can yield highly pure exosomes and is straightforward to operate, yet it is limited by high antibody costs and low yields. Microfluidics-based techniques demonstrate efficiency, but their clinical application is restricted by issues related to standardization, scalability and validation [90]. In conclusion, there is currently no single method that satisfies the criteria of high purity, high efficiency, low cost and ease of operation for clinical applications. Future research should prioritize technology optimization, the development of combined strategies and the standardization of existing technologies to facilitate their advancement toward clinical applications and large-scale production.

As an emerging treatment method for tumors, ncRNA therapeutics primarily faces three significant challenges: immunogenicity, specificity, and delivery [91]. Studies have demonstrated that opioid receptor mu (MOR) siRNA can bind to argonaute 2 (AGO2) within exosomes modified with neuron-specific rabies viral glycoprotein (RVG) peptide. The RVG exosomes can effectively pass through the blood-brain barrier by binding to acetylcholine receptors and specifically deliver MOR siRNA to Neuro2A cells rather than other non-neuronal cells, thereby reducing MOR expression levels and preventing morphine relapse. Notably, no severe immune reactions have been reported [92]. Another study developed a one-step microfluidic production method for lipid-based nanoparticles designed to mimic exosomes. The researchers found that exosome-mimicking nanoparticles modified with ITG αVβ5 significantly enhanced the efficiency of RNA delivery both *in vivo* and *in vitro*. This research not only presents a novel method for the preparation of exosome-mimicking nanoparticles but also offers valuable insights for the future development of efficient ncRNAs delivery systems [93]. Although relevant studies have made significant progress in the field of exosomal ncRNA therapy, these advancements represent only the initial steps. Future research must first achieve a deeper understanding of the delivery mechanisms and integrate the advantages of multiple disciplines, such as nanotechnology and bioengineering, with an emphasis on developing more precise and safer delivery platforms. Concurrently, it is essential to effectively address the bottlenecks in clinical translation and large-scale production to overcome these three core challenges.

The biocompatibility of exosome-based therapy is a crucial factor for its clinical translation. While exosomes are generally regarded as having good biocompatibility, potential risks arise from off-target effects and immune activation. Research indicates that exosomes derived from chromium-induced senescent L02 hepatocytes (S-L02) are enriched in miR-222-2p, which not only inhibits the proliferation of both L02 and S-L02 hepatocytes but also promotes the proliferation and migration of hepatocellular carcinoma (HCC) [94]. Another study has demonstrated that exosomes derived from packed red blood cells can activate human mast cells and promote the production of various inflammatory mediators, potentially contributing to transfusion-rel ated adverse events [95]. Although exosomes are considered highly promising carriers of ncRNAs due to their excellent biocompatibility, the associated risks cannot be overlooked. Future research should concentrate on an in-depth analysis of the mechanisms of action, optimization of targeted delivery technologies and assessment of immunogenicity to mitigate the potential ‘double-edged sword’ effect and ultimately ensure the safe and efficient clinical translation of exosome-based ncRNAs therapies.

Although exosomal ncRNAs hold significant promise for the treatment of PC, several challenges remain. These include the absence of efficient standard techniques for exosome isolation, inadequate delivery efficiency of ncRNAs and associated potential risks. Future research should prioritize the exploration of standardized, efficient, and cost-effective exosome isolation methods that are suitable for large-scale production. Additionally, it is essential to integrate multidisciplinary approaches to develop precise and safe delivery platforms. Furthermore, a deeper investigation into the mechanisms of action of exosomal ncRNAs is necessary to enhance the safety of this therapeutic strategy, ultimately facilitating clinical translation. Table 2 presents the clinical prospects of exosomal ncRNAs in PC.

**Table 2:** Clinical prospects of exosomal ncRNAs in pancreatic cancer

Clinical significance	NcRNAs	Potential role	References
**Diagnostic biomarker**	miR-191, miR-21, miR-451a	Early diagnostic and progression markers of PC and IPMN	[72]
	miR-31-5p, miR-31-3p, miR-210-3p, miR-339-5p, miR-425-5p, miR-425-3p, miR-429	Early detection of PDAC, distinguishing between chronic pancreatitis and PDAC, and monitoring high-risk patients	[73]
	miR-21, miR-10b	Early diagnosis, elevated diagnostic value, distinguishes patients with the early stage (miR-21)	[74]
	lncRNA MALAT1 and CRNDE	Higher expression in PDAC or IPMN than in healthy samples	[75]
	miR200b, miR-200c	Elevated diagnostic accuracy of PDAC after combining with CA19-9	[76]
	5 cell-free and 8 exosomal miRNAs	Identification of early-stage PDAC and CA19-9 negative cases	[77]
**Therapeutic target**	miR-485-3p	Delivered by exosomes from pancreatic ductal epithelial cells then negatively regulate PAK1 and inhibit metastasis	[80]
	miR-145-5p	Delivered by hucMSCs exosomes and inhibit PDAC cell proliferation and invasion	[81]
	miR-1231	Delivered by BM-MSC-derived exosomes and inhibit the activity of PC	[82]
	circBIRC6	Reduce circBIRC6 levels by using ASO or combining with olaparib then improve chemotherapeutic response	[83]
**Prognostic biomarker**	miR-4525, miR-451a, miR-21	Identification of poor prognosis in post-surgical PDAC patients	[86]
	miR-451a	Recurrence and poor prognosis, independent prognostic factors of OS and DFS in PDAC	[87]
	6 miRNA panel (miR-130b-5p, miR133a-3p, miR-195-5p, miR-432-5p, miR-1229-3p and miR1273f)	Evaluating whether to continue chemotherapy or surgery after NAT, predicting recurrence after surgery, serving as an independent prognostic factor for RFS, enhancing accuracy of PDAC prognosis predictions after combining with CA19-9	[88]
	circ-IRAS	Positively correlating with tumor metastasis and negatively correlating with patient survival duration	[89]

Note: ASO, antisense oligonucleotide; BM-MSCs, bone marrow mesenchymal stem cells; DFS, disease-free survival; hucMSCs, human umbilical cord-derived mesenchymal stromal cells; IPMN, intraductal papillary mucinous neoplasm; NAT, neoadjuvant therapy; PAK1, p21-activated kinase-1; PC, pancreatic cancer; PDAC, pancreatic ductal adenocarcinoma; OS, overall survival; RFS, recurrence-free survival.

## 7 Conclusion and Perspective

The role and potential of exosomal ncRNAs in PC have attracted considerable interest in recent years. Studies have demonstrated that exosomal ncRNAs play a significant role in various processes associated with malignant progression, including tumorigenesis, metastasis, and chemoresistance in PC. This occurs through the mediation of intercellular communication, regulation of related signaling pathways, and influence on the TME. Such findings offer new insights and potential targets for PC treatment. Furthermore, exosomal ncRNAs exhibit high stability, expression specificity, and ease of detection in body fluids, positioning them as a promising liquid biopsy marker for PC. This paper reviews the biological characteristics of exosomal ncRNAs, their mechanisms in PC, and their prospects for clinical application.

In terms of diagnosis and prognosis assessment, exosomal ncRNAs offer several advantages, including non-invasiveness, high sensitivity, and high specificity. The detection of exosomal ncRNAs expression levels in body fluids can dynamically reflect the staging and prognosis of PC in real time. Moreover, when combined with traditional tumor markers, it can significantly enhance the accuracy of early diagnosis and prognosis prediction for PC. In terms of treatment, exosomal ncRNAs can target relevant signaling pathways to inhibit the progression of PC. Additionally, exosomes can serve as carriers to deliver ncRNAs, thereby interfering with PC progression and enhancing drug sensitivity.

However, several noteworthy challenges remain in the current clinical application of exosomal ncRNAs. First, research into the mechanisms of exosomal ncRNAs in PC is still in its initial phases. Although the regulatory effects of various exosomal ncRNAs on PC have been identified, further research is necessary to explore the functions and expression of additional unknown exosomal ncRNAs within the complex TME of PC. Furthermore, the purification and detection methods for exosomal ncRNAs have yet to be standardized and continue to face multiple challenges, such as rapidity, stability, and cost-effectiveness, which limit their application in large-scale clinical settings. Additionally, improving the sensitivity, specificity, and diagnostic accuracy of exosomal ncRNAs in PC represents an urgent challenge that must be addressed.

To tackle these issues, upcoming studies should concentrate on several important aspects: First, a comprehensive analysis of the biological mechanisms underlying exosomal ncRNAs in PC will provide a theoretical foundation for clinical diagnosis, treatment, and prognosis assessment. Second, it is essential to develop standardized, cost-effective, and efficient purification and detection methods to provide reliable technical support for exosomal ncRNAs, enabling their potential as new biomarkers and therapeutic targets in clinical applications. Finally, additional clinical experiments are necessary to validate the diagnostic and prognostic value, as well as the therapeutic effects, of exosomal ncRNAs, thereby facilitating their translation into clinical applications.

In short, exosomal ncRNAs have emerged as a promising target in the clinical application of PC. Despite existing research barriers, we are optimistic that as investigations progress, exosomal ncRNAs will become increasingly important in the diagnosis and treatment of PC, ultimately leading to significant improvements in patient prognosis and quality of life.

## Data Availability

Data sharing not applicable to this article as no datasets were generated or analyzed during the current study.

## References

[1] Siegel RL, Giaquinto AN, Jemal A. Cancer statistics. CA Cancer J Clin. 2024;74(1):12–49; 38230766 10.3322/caac.21820

[2] Mizrahi JD, Surana R, Valle JW, Shroff RT. Pancreatic cancer. Lancet. 2020;395(10242):2008–20. doi:10.1016/s0140-6736(20)30974-0; 32593337

[3] Yuan C, Kim J, Wang QL, Lee AA, Babic A, Amundadottir LT, et al. The age-dependent association of risk factors with pancreatic cancer. Ann Oncol. 2022;33(7):693–701; 35398288 10.1016/j.annonc.2022.03.276 PMC9233063

[4] Hu ZI, O’Reilly EM. Therapeutic developments in pancreatic cancer. Nat Rev Gastroenterol Hepatol. 2024;21(1):7–24. doi:10.1038/s41575-023-00840-w; 37798442

[5] Kalluri R, LeBleu VS. The biology, function, and biomedical applications of exosomes. Science. 2020;367(6478):eaau6977; 32029601 10.1126/science.aau6977 PMC7717626

[6] Beňačka R, Szabóová D, Guľašová Z, Hertelyová Z, Radoňak J. Non-coding RNAs in human cancer and other diseases: overview of the diagnostic potential. Int J Mol Sci. 2023;24(22):16213. doi:10.3390/ijms242216213; 38003403 PMC10671391

[7] Xie Y, Dang W, Zhang S, Yue W, Yang L, Zhai X, et al. The role of exosomal noncoding RNAs in cancer. Mol Cancer. 2019;18(1):37. doi:10.1186/s12943-019-0984-4; 30849983 PMC6408816

[8] Deng C, Huo M, Chu H, Zhuang X, Deng G, Li W, et al. Exosome circATP8A1 induces macrophage M2 polarization by regulating the miR-1-3p/STAT6 axis to promote gastric cancer progression. Mol Cancer. 2024;23(1):49. doi:10.1186/s12943-024-01966-4; 38459596 PMC10921793

[9] Singh R, Pochampally R, Watabe K, Lu Z, Mo YY. Exosome-mediated transfer of miR-10b promotes cell invasion in breast cancer. Mol Cancer. 2014;13(1):256. doi:10.1186/1476-4598-13-256; 25428807 PMC4258287

[10] Chang J, Zhang L, Li Z, Qian C, Du J. Exosomal non-coding RNAs (ncRNAs) as potential biomarkers in tumor early diagnosis. Biochim Biophys Acta Rev Cancer. 2024;1879(6):189188. doi:10.1016/j.bbcan.2024.189188; 39313040

[11] Johnstone RM, Adam M, Hammond JR, Orr L, Turbide C. Vesicle formation during reticulocyte maturation. Association of plasma membrane activities with released vesicles (exosomes). J Biol Chem. 1987;262(19):9412–20. doi:10.1016/s0021-9258(18)48095-7. 3597417

[12] Huotari J, Helenius A. Endosome maturation. Embo J. 2011;30(17):3481–500. doi:10.1038/emboj.2011.286; 21878991 PMC3181477

[13] Baietti MF, Zhang Z, Mortier E, Melchior A, Degeest G, Geeraerts A, et al. Syndecan-syntenin-ALIX regulates the biogenesis of exosomes. Nat Cell Biol. 2012;14(7):677–85. doi:10.1038/ncb2502; 22660413

[14] Colombo M, Moita C, van Niel G, Kowal J, Vigneron J, Benaroch P, et al. Analysis of ESCRT functions in exosome biogenesis, composition and secretion highlights the heterogeneity of extracellular vesicles. J Cell Sci. 2013;126(Pt 24):5553–65. doi:10.1242/jcs.128868; 24105262

[15] Minakawa T, Yamashita JK. Versatile extracellular vesicle-mediated information transfer: intercellular synchronization of differentiation and of cellular phenotypes, and future perspectives. Inflamm Regen. 2024;44(1):4. doi:10.1186/s41232-024-00318-5; 38225584 PMC10789073

[16] Li Y, Zhao W, Wang Y, Wang H, Liu S. Extracellular vesicle-mediated crosstalk between pancreatic cancer and stromal cells in the tumor microenvironment. J Nanobiotechnol. 2022;20(1):208. doi:10.1186/s12951-022-01382-0; 35501802 PMC9063273

[17] Chen SW, Zhu SQ, Pei X, Qiu BQ, Xiong D, Long X, et al. Cancer cell-derived exosomal circUSP7 induces CD8(+) T cell dysfunction and anti-PD1 resistance by regulating the miR-934/SHP2 axis in NSCLC. Mol Cancer. 2021;20(1):144. doi:10.1186/s12943-021-01448-x; 34753486 PMC8576933

[18] He Z, Zhong Y, Regmi P, Lv T, Ma W, Wang J, et al. Exosomal long non-coding RNA TRPM2-AS promotes angiogenesis in gallbladder cancer through interacting with PABPC1 to activate NOTCH1 signaling pathway. Mol Cancer. 2024;23(1):65. doi:10.1186/s12943-024-01979-z; 38532427 PMC10967197

[19] Xia W, Chen W, Ni C, Meng X, Wu J, Yang Q, et al. Chemotherapy-induced exosomal circBACH1 promotes breast cancer resistance and stemness via miR-217/G3BP2 signaling pathway. Breast Cancer Res. 2023;25(1):85. doi:10.1186/s13058-023-01672-x; 37461019 PMC10351125

[20] Mo Y, Leung LL, Mak CSL, Wang X, Chan WS, Hui LMN, et al. Tumor-secreted exosomal miR-141 activates tumor-stroma interactions and controls premetastatic niche formation in ovarian cancer metastasis. Mol Cancer. 2023;22(1):4. doi:10.1186/s12943-022-01703-9; 36624516 PMC9827705

[21] Villarroya-Beltri C, Gutiérrez-Vázquez C, Sánchez-Cabo F, Pérez-Hernández D, Vázquez J, Martin-Cofreces N, et al. Sumoylated hnRNPA2B1 controls the sorting of miRNAs into exosomes through binding to specific motifs. Nat Commun. 2013;4(1):2980. doi:10.1038/ncomms3980; 24356509 PMC3905700

[22] Segura E, Guérin C, Hogg N, Amigorena S, Théry C. CD8+ dendritic cells use LFA-1 to capture MHC-peptide complexes from exosomes *in vivo*. J Immunol. 2007;179(3):1489–96. doi:10.4049/jimmunol.179.3.1489; 17641014

[23] He L, Hannon GJ. MicroRNAs: small RNAs with a big role in gene regulation. Nat Rev Genet. 2004;5(7):522–31. doi:10.1038/nrg1379; 15211354

[24] Schmitt AM, Chang HY. Long noncoding RNAs in cancer pathways. Cancer Cell. 2016;29(4):452–63. doi:10.1016/j.ccell.2016.03.010; 27070700 PMC4831138

[25] Kristensen LS, Hansen TB, Venø MT, Kjems J. Circular RNAs in cancer: opportunities and challenges in the field. Oncogene. 2018;37(5):555–65. doi:10.1038/onc.2017.361; 28991235 PMC5799710

[26] Huang J, Gao G, Ge Y, Liu J, Cui H, Zheng R, et al. Development of a serum-based MicroRNA signature for early detection of pancreatic cancer: a multicenter cohort study. Dig Dis Sci. 2024;69(4):1263–73. doi:10.1007/s10620-024-08338-4; 38451429 PMC11026211

[27] Ye Z, Zhu Z, Xie J, Feng Z, Li Y, Xu X, et al. Hsa_circ_0000069 knockdown inhibits tumorigenesis and exosomes with downregulated hsa_circ_0000069 suppress malignant transformation via inhibition of STIL in pancreatic cancer. Int J Nanomed. 2020;15:9859–73. 10.2147/IJN.S279258 PMC7732169 33324055

[28] Lin J, Wang X, Zhai S, Shi M, Peng C, Deng X, et al. Hypoxia-induced exosomal circPDK1 promotes pancreatic cancer glycolysis via c-myc activation by modulating miR-628-3p/BPTF axis and degrading BIN1. J Hematol Oncol. 2022;15(1):128. doi:10.1186/s13045-022-01348-7; 36068586 PMC9450374

[29] Li Z, Yanfang W, Li J, Jiang P, Peng T, Chen K, et al. Tumor-released exosomal circular RNA PDE8A promotes invasive growth via the miR-338/MACC1/MET pathway in pancreatic cancer. Cancer Lett. 2018;432:237–50. doi:10.1016/j.canlet.2018.04.035; 29709702

[30] Chen YY, Jiang MJ, Tian L. Analysis of exosomal circRNAs upon irradiation in pancreatic cancer cell repopulation. BMC Med Genomics. 2020;13(1):107. doi:10.1186/s12920-020-00756-3; 32727565 PMC7391519

[31] Thiery JP, Acloque H, Huang RY, Nieto MA. Epithelial-mesenchymal transitions in development and disease. Cell. 2009;139(5):871–90. doi:10.1016/j.cell.2009.11.007; 19945376

[32] Sun Z, Sun D, Feng Y, Zhang B, Sun P, Zhou B, et al. Exosomal linc-ROR mediates crosstalk between cancer cells and adipocytes to promote tumor growth in pancreatic cancer. Mol Ther Nucleic Acids. 2021;26:253–68. doi:10.1016/j.omtn.2021.06.001; 34513308 PMC8413664

[33] Wang X, Li H, Lu X, Wen C, Huo Z, Shi M, et al. Melittin-induced long non-coding RNA NONHSAT105177 inhibits proliferation and migration of pancreatic ductal adenocarcinoma. Cell Death Dis. 2018;9(10):940. doi:10.1038/s41419-018-0965-3; 30237397 PMC6148000

[34] Li Z, Jiang P, Li J, Peng M, Zhao X, Zhang X, et al. Tumor-derived exosomal lnc-Sox2ot promotes EMT and stemness by acting as a ceRNA in pancreatic ductal adenocarcinoma. Oncogene. 2018;37(28):3822–38. doi:10.1038/s41388-018-0237-9; 29643475

[35] Ma Q, Wu H, Xiao Y, Liang Z, Liu T. Upregulation of exosomal microRNA-21 in pancreatic stellate cells promotes pancreatic cancer cell migration and enhances Ras/ERK pathway activity. Int J Oncol. 2020;56(4):1025–33; 32319558 10.3892/ijo.2020.4986

[36] Mikamori M, Yamada D, Eguchi H, Hasegawa S, Kishimoto T, Tomimaru Y, et al. MicroRNA-155 controls exosome synthesis and promotes gemcitabine resistance in pancreatic ductal adenocarcinoma. Sci Rep. 2017;7(1):42339. doi:10.1038/srep42339; 28198398 PMC5309735

[37] Binenbaum Y, Fridman E, Yaari Z, Milman N, Schroeder A, Ben David G, et al. Transfer of miRNA in macrophage-derived exosomes induces drug resistance in pancreatic adenocarcinoma. Cancer Res. 2018;78(18):5287–99. doi:10.1158/0008-5472.can-18-0124; 30042153

[38] Yang Z, Zhao N, Cui J, Wu H, Xiong J, Peng T. Exosomes derived from cancer stem cells of gemcitabine-resistant pancreatic cancer cells enhance drug resistance by delivering miR-210. Cell Oncol. 2020;43(1):123–36. doi:10.1007/s13402-019-00476-6; 31713003 PMC12990725

[39] Zeng Z, Zhao Y, Chen Q, Zhu S, Niu Y, Ye Z, et al. Hypoxic exosomal HIF-1α-stabilizing circZNF91 promotes chemoresistance of normoxic pancreatic cancer cells via enhancing glycolysis. Oncogene. 2021;40(36):5505–17. doi:10.1038/s41388-021-01960-w; 34294845

[40] Wang H, Min J, Xu C, Liu Y, Yu Z, Gong A, et al. Hypoxia-elicited exosomes promote the chemoresistance of pancreatic cancer cells by transferring LncROR via hippo signaling. J Cancer. 2023;14(6):1075–87. doi:10.7150/jca.81320; 37151398 PMC10158512

[41] Di Pace AL, Pelosi A, Fiore PF, Tumino N, Besi F, Quatrini L, et al. MicroRNA analysis of Natural Killer cell-derived exosomes: the microRNA let-7b-5p is enriched in exosomes and participates in their anti-tumor effects against pancreatic cancer cells. Oncoimmunology. 2023;12(1):2221081. doi:10.1080/2162402x.2023.2221081; 37304055 PMC10251800

[42] Shi X, Pang S, Zhou J, Yan G, Gao R, Wu H, et al. Bladder-cancer-derived exosomal circRNA_0013936 promotes suppressive immunity by up-regulating fatty acid transporter protein 2 and down-regulating receptor-interacting protein kinase 3 in PMN-MDSCs. Mol Cancer. 2024;23(1):52. doi:10.1186/s12943-024-01968-2; 38461272 PMC10924381

[43] Sun Z, Xu Y, Shao B, Dang P, Hu S, Sun H, et al. Exosomal circPOLQ promotes macrophage M2 polarization via activating IL-10/STAT3 axis in a colorectal cancer model. J Immunother Cancer. 2024;12(5):e008491. doi:10.1136/jitc-2023-008491; 38782541 PMC11116870

[44] Zhou M, Chen J, Zhou L, Chen W, Ding G, Cao L. Pancreatic cancer derived exosomes regulate the expression of TLR4 in dendritic cells via miR-203. Cell Immunol. 2014;292(1–2):65–9. doi:10.1016/j.cellimm.2014.09.004; 25290620

[45] Yao H, Huang C, Zou J, Liang W, Zhao Y, Yang K, et al. Extracellular vesicle-packaged lncRNA from cancer-associated fibroblasts promotes immune evasion by downregulating HLA-A in pancreatic cancer. J Extracell Vesicles. 2024;13(7):e12484; 39041344 10.1002/jev2.12484 PMC11263977

[46] Chen C, Liu Y, Liu L, Si C, Xu Y, Wu X, et al. Exosomal circTUBGCP4 promotes vascular endothelial cell tipping and colorectal cancer metastasis by activating Akt signaling pathway. J Exp Clin Cancer Res. 2023;42(1):46. doi:10.1186/s13046-023-02619-y; 36793126 PMC9930311

[47] Zeng Z, Li Y, Pan Y, Lan X, Song F, Sun J, et al. Cancer-derived exosomal miR-25-3p promotes pre-metastatic niche formation by inducing vascular permeability and angiogenesis. Nat Commun. 2018;9(1):5395. doi:10.1038/s41467-018-07810-w; 30568162 PMC6300604

[48] Chen K, Wang Q, Liu X, Wang F, Yang Y, Tian X. Hypoxic pancreatic cancer derived exosomal miR-30b-5p promotes tumor angiogenesis by inhibiting GJA1 expression. Int J Biol Sci. 2022;18(3):1220–37. doi:10.7150/ijbs.67675; 35173549 PMC8771853

[49] Guo Z, Wang X, Yang Y, Chen W, Zhang K, Teng B, et al. Hypoxic tumor-derived exosomal long noncoding RNA UCA1 promotes angiogenesis via miR-96-5p/AMOTL2 in pancreatic cancer. Mol Ther Nucleic Acids. 2020;22:179–95. doi:10.1016/j.omtn.2020.08.021; 32942233 PMC7498711

[50] Liu Y, Cao X. Characteristics and significance of the pre-metastatic niche. Cancer Cell. 2016;30(5):668–81; 27846389 10.1016/j.ccell.2016.09.011

[51] Sun H, Meng Q, Shi C, Yang H, Li X, Wu S, et al. Hypoxia-inducible exosomes facilitate liver-tropic premetastatic niche in colorectal cancer. Hepatology. 2021;74(5):2633–51. doi:10.1002/hep.32009; 34110633

[52] Rana S, Malinowska K, Zöller M. Exosomal tumor microRNA modulates premetastatic organ cells. Neoplasia. 2013;15(3):281–95. doi:10.1593/neo.122010; 23479506 PMC3593151

[53] Chen W, Peng W, Wang R, Bai S, Cao M, Xiong S, et al. Exosome-derived tRNA fragments tRF-GluCTC-0005 promotes pancreatic cancer liver metastasis by activating hepatic stellate cells. Cell Death Dis. 2024;15(1):102. doi:10.1038/s41419-024-06482-3; 38291031 PMC10827722

[54] Zheng S, Hu C, Lin Q, Li T, Li G, Tian Q, et al. Extracellular vesicle-packaged PIAT from cancer-associated fibroblasts drives neural remodeling by mediating m5C modification in pancreatic cancer mouse models. Sci Transl Med. 2024;16(756):eadi0178. doi:10.1126/scitranslmed.adi0178; 39018369

[55] Ren J, Ding L, Zhang D, Shi G, Xu Q, Shen S, et al. Carcinoma-associated fibroblasts promote the stemness and chemoresistance of colorectal cancer by transferring exosomal lncRNA H19. Theranostics. 2018;8(14):3932–48. doi:10.7150/thno.25541; 30083271 PMC6071523

[56] Zhang H, Deng T, Liu R, Ning T, Yang H, Liu D, et al. CAF secreted miR-522 suppresses ferroptosis and promotes acquired chemo-resistance in gastric cancer. Mol Cancer. 2020;19(1):43. doi:10.1186/s12943-020-01168-8; 32106859 PMC7045485

[57] Chen F, Chen J, Yang L, Liu J, Zhang X, Zhang Y, et al. Extracellular vesicle-packaged HIF-1α-stabilizing lncRNA from tumour-associated macrophages regulates aerobic glycolysis of breast cancer cells. Nat Cell Biol. 2019;21(4):498–510. doi:10.1038/s41556-019-0299-0; 30936474

[58] Chhabra Y, Weeraratna AT. Fibroblasts in cancer: unity in heterogeneity. Cell. 2023;186(8):1580–609. doi:10.1016/j.cell.2023.03.016; 37059066 PMC11422789

[59] Masamune A, Yoshida N, Hamada S, Takikawa T, Nabeshima T, Shimosegawa T. Exosomes derived from pancreatic cancer cells induce activation and profibrogenic activities in pancreatic stellate cells. Biochem Biophys Res Commun. 2018;495(1):71–7. doi:10.1016/s0016-5085(17)30945-9. 29111329

[60] Dixon SJ, Lemberg KM, Lamprecht MR, Skouta R, Zaitsev EM, Gleason CE, et al. Ferroptosis: an iron-dependent form of nonapoptotic cell death. Cell. 2012;149(5):1060–72. doi:10.1016/j.cell.2012.03.042; 22632970 PMC3367386

[61] Chen X, Kang R, Kroemer G, Tang D. Broadening horizons: the role of ferroptosis in cancer. Nat Rev Clin Oncol. 2021;18(5):280–96. doi:10.1038/s41571-020-00462-0; 33514910

[62] Doll S, Proneth B, Tyurina YY, Panzilius E, Kobayashi S, Ingold I, et al. ACSL4 dictates ferroptosis sensitivity by shaping cellular lipid composition. Nat Chem Biol. 2017;13(1):91–8. doi:10.1038/nchembio.2239; 27842070 PMC5610546

[63] Qi R, Bai Y, Li K, Liu N, Xu Y, Dal E, et al. Cancer-associated fibroblasts suppress ferroptosis and induce gemcitabine resistance in pancreatic cancer cells by secreting exosome-derived ACSL4-targeting miRNAs. Drug Resist Updat. 2023;68:100960. doi:10.1016/j.drup.2023.100960; 37003125

[64] Faubert B, Solmonson A, DeBerardinis RJ. Metabolic reprogramming and cancer progression. Science. 2020;368(6487):eaaw5473. doi:10.1126/science.aaw5473; 32273439 PMC7227780

[65] Suzuki T, Otsuka M, Seimiya T, Iwata T, Kishikawa T, Koike K. The biological role of metabolic reprogramming in pancreatic cancer. MedComm. 2020;1(3):302–10. doi:10.1002/mco2.37; 34766124 PMC8491225

[66] Zhang P, Wang Q, Lu W, Zhang F, Wu D, Sun J. NNT-AS1 in CAFs-derived exosomes promotes progression and glucose metabolism through miR-889-3p/HIF-1α in pancreatic adenocarcinoma. Sci Rep. 2024;14(1):6979. doi:10.1038/s41598-024-57769-6; 38521881 PMC10960871

[67] Toledo FGS, Chari S, Yadav D. Understanding the contribution of insulin resistance to the risk of pancreatic cancer. Am J Gastroenterol. 2021;116(4):669–70. doi:10.14309/ajg.0000000000001104; 33982933 PMC8178512

[68] Zhang AMY, Xia YH, Lin JSH, Chu KH, Wang WCK, Ruiter TJJ, et al. Hyperinsulinemia acts via acinar insulin receptors to initiate pancreatic cancer by increasing digestive enzyme production and inflammation. Cell Metab. 2023;35(12):2119–35.e5. doi:10.1016/j.cmet.2023.10.003; 37913768

[69] Wang L, Zhang B, Zheng W, Kang M, Chen Q, Qin W, et al. Exosomes derived from pancreatic cancer cells induce insulin resistance in C2C12 myotube cells through the PI3K/Akt/FoxO1 pathway. Sci Rep. 2017;7(1):5384. doi:10.1038/s41598-017-05541-4; 28710412 PMC5511275

[70] Guo X, Peng Y, Song Q, Wei J, Wang X, Ru Y, et al. A liquid biopsy signature for the early detection of gastric cancer in patients. Gastroenterology. 2023;165(2):402–13.e13. doi:10.1053/j.gastro.2023.02.044; 36894035

[71] Ghosh S, Bhowmik S, Majumdar S, Goswami A, Chakraborty J, Gupta S, et al. The exosome encapsulated microRNAs as circulating diagnostic marker for hepatocellular carcinoma with low alpha-fetoprotein. Int J Cancer. 2020;147(10):2934–47. doi:10.1002/ijc.33111; 32441313

[72] Goto T, Fujiya M, Konishi H, Sasajima J, Fujibayashi S, Hayashi A, et al. An elevated expression of serum exosomal microRNA-191, -21, -451a of pancreatic neoplasm is considered to be efficient diagnostic marker. BMC Cancer. 2018;18(1):116. doi:10.1186/s12885-018-4006-5; 29385987 PMC5793347

[73] Makler A, Narayanan R, Asghar W. An exosomal miRNA biomarker for the detection of pancreatic ductal adenocarcinoma. Biosensors. 2022;12(10):831. doi:10.3390/bios12100831; 36290970 PMC9599289

[74] Pu X, Ding G, Wu M, Zhou S, Jia S, Cao L. Elevated expression of exosomal microRNA-21 as a potential biomarker for the early diagnosis of pancreatic cancer using a tethered cationic lipoplex nanoparticle biochip. Oncol Lett. 2020;19(3):2062–70; 32194703 10.3892/ol.2020.11302 PMC7039151

[75] Kumar SR, Kimchi ET, Manjunath Y, Gajagowni S, Stuckel AJ, Kaifi JT. RNA cargos in extracellular vesicles derived from blood serum in pancreas associated conditions. Sci Rep. 2020;10(1):2800. doi:10.1038/s41598-020-59523-0; 32071328 PMC7028741

[76] Reese M, Flammang I, Yang Z, Dhayat SA. Potential of exosomal microRNA-200b as liquid biopsy marker in pancreatic ductal adenocarcinoma. Cancers. 2020;12(1):197. doi:10.3390/cancers12010197; 31941049 PMC7016821

[77] Nakamura K, Zhu Z, Roy S, Jun E, Han H, Munoz RM, et al. An exosome-based transcriptomic signature for noninvasive, early detection of patients with pancreatic ductal adenocarcinoma: a multicenter cohort study. Gastroenterology. 2022;163(5):1252–66.e2. doi:10.1053/j.gastro.2022.06.090; 35850192 PMC9613527

[78] Taghikhani A, Hassan ZM, Ebrahimi M, Moazzeni SM. microRNA modified tumor-derived exosomes as novel tools for maturation of dendritic cells. J Cell Physiol. 2019;234(6):9417–27. doi:10.1002/jcp.27626; 30362582

[79] Guo Z, Zhang Y, Xu W, Zhang X, Jiang J. Engineered exosome-mediated delivery of circDIDO1 inhibits gastric cancer progression via regulation of MiR-1307-3p/SOCS2 Axis. J Transl Med. 2022;20(1):326. doi:10.1186/s12967-022-03527-z; 35864511 PMC9306104

[80] Li M, Zhou J, Zhang Z, Li J, Wang F, Ma L, et al. Exosomal miR-485-3p derived from pancreatic ductal epithelial cells inhibits pancreatic cancer metastasis through targeting PAK1. Chin Med J. 2022;135(19):2326–37. doi:10.1097/cm9.0000000000002154; 36535010 PMC9771326

[81] Ding Y, Cao F, Sun H, Wang Y, Liu S, Wu Y, et al. Exosomes derived from human umbilical cord mesenchymal stromal cells deliver exogenous miR-145-5p to inhibit pancreatic ductal adenocarcinoma progression. Cancer Lett. 2019;442:351–61. doi:10.1016/j.canlet.2018.10.039; 30419348

[82] Shang S, Wang J, Chen S, Tian R, Zeng H, Wang L, et al. Exosomal miRNA-1231 derived from bone marrow mesenchymal stem cells inhibits the activity of pancreatic cancer. Cancer Med. 2019;8(18):7728–40. doi:10.1002/cam4.2633; 31642612 PMC6912060

[83] Zheng S, Tian Q, Yuan Y, Sun S, Li T, Xia R, et al. Extracellular vesicle-packaged circBIRC6 from cancer-associated fibroblasts induce platinum resistance via SUMOylation modulation in pancreatic cancer. J Exp Clin Cancer Res. 2023;42(1):324. doi:10.1186/s13046-023-02854-3; 38012734 PMC10683239

[84] Guo T, Tang XH, Gao XY, Zhou Y, Jin B, Deng ZQ, et al. A liquid biopsy signature of circulating exosome-derived mRNAs, miRNAs and lncRNAs predict therapeutic efficacy to neoadjuvant chemotherapy in patients with advanced gastric cancer. Mol Cancer. 2022;21(1):216. doi:10.1186/s12943-022-01684-9; 36510184 PMC9743536

[85] Wang X, Qian T, Bao S, Zhao H, Chen H, Xing Z, et al. Circulating exosomal miR-363-5p inhibits lymph node metastasis by downregulating PDGFB and serves as a potential noninvasive biomarker for breast cancer. Mol Oncol. 2021;15(9):2466–79. doi:10.1002/1878-0261.13029; 34058065 PMC8410538

[86] Kawamura S, Iinuma H, Wada K, Takahashi K, Minezaki S, Kainuma M, et al. Exosome-encapsulated microRNA-4525, microRNA-451a and microRNA-21 in portal vein blood is a high-sensitive liquid biomarker for the selection of high-risk pancreatic ductal adenocarcinoma patients. J Hepatobiliary Pancreat Sci. 2019;26(2):63–72. doi:10.1002/jhbp.601; 30561106

[87] Takahasi K, Iinuma H, Wada K, Minezaki S, Kawamura S, Kainuma M, et al. Usefulness of exosome-encapsulated microRNA-451a as a minimally invasive biomarker for prediction of recurrence and prognosis in pancreatic ductal adenocarcinoma. J Hepatobiliary Pancreat Sci. 2018;25(2):155–61. doi:10.1002/jhbp.524; 29130611

[88] Nishiwada S, Cui Y, Sho M, Jun E, Akahori T, Nakamura K, et al. Transcriptomic profiling identifies an exosomal microRNA signature for predicting recurrence following surgery in patients with pancreatic ductal adenocarcinoma. Ann Surg. 2022;276(6):e876–e85. doi:10.1097/sla.0000000000004993; 34132691 PMC8674379

[89] Li J, Li Z, Jiang P, Peng M, Zhang X, Chen K, et al. Circular RNA IARS (circ-IARS) secreted by pancreatic cancer cells and located within exosomes regulates endothelial monolayer permeability to promote tumor metastasis. J Exp Clin Cancer Res. 2018;37(1):177. doi:10.1186/s13046-018-0822-3; 30064461 PMC6069563

[90] Yang D, Zhang W, Zhang H, Zhang F, Chen L, Ma L, et al. Progress, opportunity, and perspective on exosome isolation—efforts for efficient exosome-based theranostics. Theranostics. 2020;10(8):3684–707. doi:10.7150/thno.41580; 32206116 PMC7069071

[91] Winkle M, El-Daly SM, Fabbri M, Calin GA. Noncoding RNA therapeutics—challenges and potential solutions. Nat Rev Drug Discov. 2021;20(8):629–51. doi:10.1038/s41573-021-00219-z; 34145432 PMC8212082

[92] Liu Y, Li D, Liu Z, Zhou Y, Chu D, Li X, et al. Targeted exosome-mediated delivery of opioid receptor Mu siRNA for the treatment of morphine relapse. Sci Rep. 2015;5(1):17543. doi:10.1038/srep17543; 26633001 PMC4668387

[93] Maeki M, Niwa A, Oyama S, Aratani K, Ito R, Suzuki Y, et al. Microfluidic production of exosome-mimicking lipid nanoparticles for enhanced RNA delivery: role of exosomal proteins. ACS Appl Mater Interfaces. 2025;17(29):41666–79. doi:10.1021/acsami.5c06927; 40521777 PMC12292319

[94] Ma Y, Li S, Ye S, Luo S, Wei L, Su Y, et al. The role of miR-222-2p in exosomes secreted by hexavalent chromium-induced premature senescent hepatocytes as a SASP component. Environ Pollut. 2024;346(17):123535. doi:10.1016/j.envpol.2024.123535; 38365080

[95] Fang X, Li J, Hao X, Zhang W, Zhong J, Zhu T, et al. Exosomes from packed red cells induce human mast cell activation and the production of multiple inflammatory mediators. Front Immunol. 2021;12:677905. doi:10.3389/fimmu.2021.677905; 34025676 PMC8135094

